# Knowledge translation strategies for sharing evidence-based health information with older adults and their caregivers: findings from a persona-scenario method

**DOI:** 10.1186/s12877-021-02588-x

**Published:** 2021-11-23

**Authors:** Cynthia Lokker, Stephen J. Gentles, Rebecca Ganann, Rita Jezrawi, Irtaza Tahir, Opeyemi Okelana, Claudia Yousif, Alfonso Iorio, Ruta Valaitis

**Affiliations:** 1 Department of Health Research Methods, Evidence, and Impact, McMaster University, Hamilton, ON Canada; 2 Department of Psychiatry and Behavioural Neurosciences, McMaster University, Hamilton, ON Canada; 3 School of Nursing, McMaster University, Hamilton, ON Canada; 4 Temerty Faculty of Medicine, University of Toronto, Toronto, ON Canada; 5 Department of Occupational Science & Occupational Therapy, University of Toronto, Mississauga, ON Canada; 6 Department of Health Research Methods, Evidence, and Impact, Department of Medicine, McMaster University, Hamilton, ON Canada

**Keywords:** Knowledge translation, Information seeking behavior, Aged, Older adults, Healthy

## Abstract

**Background:**

By understanding the information seeking behaviors of older adults, we can better develop or iterate effective information technologies, such as the McMaster Optimal Aging Portal, that provide evidence-based health information to the public. This paper reports health-related information seeking and searching behaviours and provides strategies for effective knowledge translation (KT) to increase awareness and use of reliable health information.

**Methods:**

We conducted a qualitative study with eighteen older adults using the persona-scenario method, whereby participants created personas and scenarios describing older adults seeking health information. Scenarios were analyzed using a two-phase inductive qualitative approach, with the personas as context. From the findings related to pathways of engaging with health information, we identified targeted KT strategies to raise awareness and uptake of evidence-based information resources.

**Results:**

Twelve women and six men, 60 to 81 years of age, participated. In pairs, they created twelve personas that captured rural and urban, male and female, and immigrant perspectives. Some scenarios described older adults who did not engage directly with technology, but rather accessed information indirectly through other sources or preferred nondigital modes of delivery. Two major themes regarding KT considerations were identified: *connecting to information via other people and personal venues* (people included healthcare professionals, librarians, and personal networks; personal venues included clinics, libraries, pharmacies, and community gatherings); and *health information delivery formats*, (e.g., printed and multimedia formats for web-based resources). For each theme, and any identified subthemes, corresponding sets of suggested KT strategies are presented.

**Conclusions:**

Our findings underline the importance of people, venues, and formats in the actions of older adults seeking trusted health information and highlight the need for enhanced KT strategies to share information across personal and professional networks of older adults. KT strategies that could be employed by organizations or communities sharing evidence-based, reliable health information include combinations of educational outreach and materials, decision support tools, small group sessions, publicity campaigns, champions/opinion leaders, and conferences.

**Supplementary Information:**

The online version contains supplementary material available at 10.1186/s12877-021-02588-x.

## Background

Older adults (≥60 years) seek health information for a variety of reasons, including to inform themselves about prognoses, treatment options, and symptoms; learn about a recent diagnosis or treatment of a new medical issue; manage or cope with their chronic condition; and clarify information provided by their healthcare professionals [1–3]. To address these information needs, this population is increasingly seeking health information on the internet [1, 4–6]. Determinants of internet use by older adults include having trust in health information websites and the perceived usefulness of the internet as a health information source [7]. While older adults and people with chronic conditions have a relatively high demand for health information, many have low eHealth literacy and are not confident in their searching abilities [1, 2]. To meet the unique needs of older adults seeking relevant and timely Internet-based health evidence, information communication technologies (ICT) must be designed to be as accessible as possible for this population [8].

With the growing adoption of ICT to search for and share information and to facilitate communication with loved ones and healthcare providers [9], there is a need to understand older adults’ information-seeking behaviours and how to best guide them to reliable health information resources. Information behaviours encompass how a person identifies their need for information, [10] information seeking behaviours (i.e., how people discover and access information), information searching behaviours (i.e., how people otherwise interact with information and information systems, including appraisal and application of the information they encounter), and information use [10]. As information seeking and searching activities relies on cognitive abilities such as working memory and reasoning, the processes of finding relevant and credible information for those with limited knowledge or experience using the Internet and search engines can be challenging [11]. To improve digital literacy, it is necessary to expand experience and comfort with technology. It is difficult, however, to expect older adults to develop the necessary skills when the appropriate supports are not available or accessible [12]. We know that many older adults frequently rely on family, friends, and healthcare providers as their primary information resources [13–15]. However, users of online information tend to independently navigate and use digital information resources [14]. Currently, few programs or other supports exist to help older adults find and use higher quality evidence-based health information online [16].

The McMaster Optimal Aging Portal (the Portal) is a web-based resource that provides lay summaries of systematic literature reviews, evidence-based blog posts, and web resource ratings on topics relevant to health and aging for older adults [17]. Information provided on the Portal is pre-appraised by researchers who are trained to identify high quality, relevant health research information that is appropriate for wider dissemination. This information is then interpreted and presented for a range of audiences, including the public, clinicians, public health professionals, and policy makers. The Portal was developed as a knowledge translation (KT) tool, disseminating high quality information based on rigorous research findings with the goal of supporting older adults or their caregivers to adopt evidence-informed personal behaviours or practices that promote healthy aging. KT addresses “the gap between what is known from research, and implementation of this knowledge by key stakeholders with the intention of improving health outcomes and efficiencies of the health care system” [18]. Broadly, KT interventions are developed to change behaviour or practice and include a comprehensive set of strategies that range from passive dissemination of information to more active approaches through knowledge brokers [19], educational sessions, alerting features in electronic systems, and active feedback after monitoring actions [20].

By understanding the information seeking behaviors of older adults, we can better develop or iterate effective technologies that supply evidence-based health information, like the Portal, to promote their use by older adults, their caregivers, and broader audiences. To fully realize its potential for supporting older adult behavior, it is important to also “disseminate” and maximize the reach and use of information technologies like the Portal by sharing them at the point of information seeking and searching.

The value of involving patients and care providers in the design of health services, programs, and interventions is widely recognized [21]. Co-design has been defined as “collective creativity” applied to a design process to develop a product, service, or interface [22]. It is the strategy by which designers engage users of a technology or health service as full partners in the learning process that underlies its design [23] and is accepted as an effective means to ensure health interventions or programs meet user needs by increasing the relevance of design features [24, 25]. Co-designing with older adults, for example, is increasingly being used as an approach to develop health information communication technologies (ICTs) that are more acceptable, relevant, and usable by older adults (for example, [26]). While not itself a co-design approach, the persona-scenario method provides an innovative means for involving users in health services design, and has been traditionally applied to the design of ICTs [27–29].

This paper reports on a subset of findings from the first phase of an overarching three-phase project. The purpose of the three phases were to: (1) use a user-focused design approach to better understand the needs of older adults with respect to health-related information seeking and searching (this paper); (2) summarize user requirements, engage with Portal decision-makers to inform feasibility and viability, and apply phase one findings to the development of prototypes intended to facilitate the effectiveness of the Portal in promoting evidence uptake by older adults; and (3) conduct a preliminary evaluation of those prototypes.

In the first phase, we conducted a qualitative study employing the persona-scenario method to design a set of generic design specifications that could be applied to any ICT [30]. This innovative design approach engages users to inform program or resource development in a way that centralizes their perspectives when determining user requirements [31–33]. By engaging potential users in the process, ideas are not limited to those of the research and design teams [29]. Through this study, we gained insight into the ways in which older adults actively seek or passively encounter health information. This paper provides a systematic report of our findings related to these “pathways of engagement” and suggests targeted KT (implementation) strategies to raise awareness and uptake of evidence-based information resources like the Portal. Knowing where older adults look and who they trust, we have compiled a series of KT strategies supported by previous evidence [34–39]. The suggested KT strategies and products can be incorporated into multifaceted KT plans for sharing health information and resources with older adults, including through ICT.

## Methods

The persona-scenario method used in this study engages end users in semi-structured activities aimed at eliciting health promotion intervention requirements—i.e., what users want the intervention to do to meet their needs [29]. User-participants are given as much latitude as possible in determining the final program or intervention requirements within pre-specified design constraints [29, 33]. The method can also produce insights and strategies for implementing an intervention or program, such as how to best promote it to target users.

### Sampling and recruitment

Participants in the persona-scenario exercise included both older adults, and adult caregivers of older adults. To be included, all participants had to be aged 60 years or older, able to read and speak in English, and reside close enough to be able to attend a southern Ontario workshop. Reimbursement for travel and free parking was provided and personal assistance was available to participants with accessibility needs. Participants were purposefully selected according to a stratified sampling strategy to reflect diverse perspectives with respect to accessing and using health information technologies—including varying levels of comfort with technology, where they typically accessed such information (home vs. community), gender, age, and socioeconomic status. Snowball sampling was used to identify participants to satisfy unmet sampling needs. Some sampling goals were adjusted iteratively after early persona scenario sessions (e.g., we sampled more older adults with higher levels of comfort with technology after early participants created personas reflecting individuals with lower levels of comfort).

Potential participants were identified and recruited through a network of older adult research advisors, partnering sites affiliated with our university research unit, and a community newspaper article promoting the study. A research coordinator distributed information and recruitment materials to partnering sites, conducted eligibility screening, and verbally explained the consent process by telephone.

### Data collection

The persona-scenario exercise consisted of a single 2-hour session, which was held in an accessible community setting (e.g., ground level location, accessible by public transportation, free parking). In total, three sessions were held with six older adult participants in each (no participants attended more than one session). Sessions began with research team facilitators (RV, SJG, RG) introducing the structure of the session and participants’ roles. Facilitators presented structured questions outlining specific tasks required for developing a persona and a scenario (see Additional file 1: Appendix: Persona-Scenario Discussion Guide). This included instructions for constructing a realistic but hypothetical *persona*, based on their personal experiences, to authentically represent an older adult with one or more concerns or needs for trusted health-promoting information. Additionally, participants were instructed on how to construct a hypothetical scenario featuring their *persona* finding and interacting with one or more ICT sources of evidence-based health information relevant to that persona. As an important adaptation in this study, rather than introducing a single ICT (or intervention) for participants to develop their persona’s scenario around as in previous persona-scenario research [33], participants were presented with examples of multiple types of ICTs and asked to consider a full range of possible technologies older adults may use to find and interact with evidence-based health information when developing their scenarios. All sessions were completed in the winter of 2017.

Following previous recommendations [33], participants were grouped in homogenous two-person teams (i.e., having similar characteristics, such as comfort with technology). Each pair had an assigned facilitator to guide them through the process, answer questions, and take notes. Pairs generally constructed one persona and one scenario in their session; but if they had time, some pairs created a second persona and scenario. After developing their persona and scenario, each pair presented a summary to the other session participants and facilitators. These summaries were audio-recorded. Session facilitators summarized the discussed themes across personas and scenarios comparing, contrasting, and seeking clarification as a means of preliminary member checking prior to subsequent analysis.

### Analysis

 The audio-recorded persona-scenario summaries produced by each participant pair were transcribed verbatim, and analysts verified and cleaned transcripts against the recordings prior to analysis. We analyzed these transcripts alongside the related notes from each session using an inductive qualitative approach that was first descriptive and then interpretive, an approach similar to the Health TAPESTRY study [30]. The descriptive phase was focused on identifying (1) user requirements to inform the design (e.g., mode, depth of content) of any ICT to deliver evidence-based health information to older adults or their caregivers, and (2) considerations for implementing such technologies. This analysis phase involved open coding of transcripts (RG, SJG, IT, CY, OO-A) using qualitative data analysis software (NVivo11, QSR International). Code names and definitions were iteratively refined as analysis progressed. Codes were then grouped into higher order themes (categories) with broader definitions. The resulting coding structure represented a set of user requirements in varying levels of detail.

In the second analysis phase, the broader research team used an interpretive approach, reviewing the coding structure and user requirements identified in the first phase, and translating them to generalizable design specifications that could be applied to broad-ranging ICTs directed at older adults. Since the persona-scenario exercise was directed at users creating personas to access health information from any source (or ICT), and was not intervention-specific, there was no specific target intervention or program to plan actions or generate products for. Thus, the interpretive phase of the analysis in our study was directed at generating more generic *design specifications*—where KT-relevant considerations are included as an important aspect of the design—which are essentially recommendations that may be considered in the design of any technology for delivering evidence-based health information to older adults.

We employed the Knowledge Translation Planning Template [34] to map actionable KT strategies and information products geared to older adults informed by the information seeking behaviours described in the scenarios. The planning template guides knowledge creators through the steps of identifying knowledge users, the main messages to share, and the goals of the KT strategies.

### Ethical Considerations

 This study was approved by the Hamilton Integrated Research Ethics Board. Participants provided written consent, received honoraria for their time, and were reimbursed for travel and other study-related expenses.

## Results

Eighteen participants were recruited, of which twelve were women and six were men; ages ranged from 60 to 81 years. Participants indicated level of comfort with technology as low (n=7), medium (n=4), or high (n=7); 15 had some level of post-secondary education, and 17 had access to technology at home. They constructed a total of 12 personas and 11 scenarios, which represented some rural and urban, male and female, and immigrant perspectives.

Two major themes were developed from the analysis of persona scenario data regarding KT considerations: *connecting to information via other people and personal venues* where examples of people include healthcare professionals, librarians, and personal networks; and examples of personal venues include clinics, libraries, pharmacies, and community gatherings; and *health information delivery formats*, such as printed or multimedia formats for web-based resources. For each theme, and any identified subthemes, we present a corresponding set of suggested KT strategies that could include some or all of the following: (a) educational outreach/materials, (b) information technology decision support, (c) interactive small group sessions, (d) media campaign/social media, (e) champions/opinion leaders, and (f) conferences.

### Theme 1: Connecting to information via other people and personal venues

#### Subtheme: Trust in health information from healthcare provider

The scenarios indicated that older adults rely on their healthcare providers (e.g., family physicians, pharmacists) for information about their health and healthcare. For example, Mabel regularly relies on her physician for information: *“Once a week Mabel still visits the doctor to rely on his information. The doctor discusses the information and then asks his nurse to provide a handout.”* These healthcare providers are trusted sources of information, as illustrated by Frank:

*You can trust something if it’s available in a doctor’s office, but if you get it elsewhere you can be a bit skeptical, especially if it’s online… knowing who the source of the information is and does the information seem reasonable…that it should be aligned with a health care professional.*

Given the important roles that healthcare providers play as trusted sources of information, tailored KT strategies to increase awareness of the Portal and similar ICTs among providers are recommended (Table 1). The goals should be to increase knowledge and use of the ICTs by practitioners to support educating their patients.

**Table 1:** KT strategies involving healthcare providers

Theme: *Connecting to information via people and venues*	
Subtheme	Suggested KT strategies
Trust in health information from healthcare providers	**Educational outreach and materials**
	• Create a publicity campaign targeting primary care physicians and pharmacists to inform them of the utility of the Portal or similar ICTs as a source of trustworthy information for older adult patients • Provide printable handouts, posters, pamphlets with instructions to access the ICT or other form of specific health information • Distribute printed outreach materials to physician offices and pharmacies; encourage placement and availability to patients or to facilitate conversations
	**Information technology decision support**
	• Encourage clinicians to push health promotion and health information to patients through targeted emails or using ICT functionality within their health record • Enable push notifications or messages that contain a website link to access information on applicable health conditions (e.g., facts of the week), and notifications for their care plans (e.g., appointment times)
	**Champions/opinion leaders**
	• Develop relationships and connections with various health professionals to promote awareness of ICTs, including primary health care practitioners, regional health organizations, home health care and telehealth nurses, optometrists, and pharmacists, traditional healers

#### Subtheme: Librarians and libraries as knowledge hubs

Libraries were described as social and knowledge hubs for the personas, *“[Alice] often peruses the collection of pamphlets for health-related topics…if, in fact, the library doesn’t have that sort of information that would be one channel to get the information out*.” Several scenarios described roles for librarians as knowledge brokers, including: *“she heads to the library and she asks the librarian for help for information” (Edna)* and *“the usual way she has of getting trusted health information is she goes to a library to access internet, and to read books, access magazines, get help from the librarian…” (Sally).* Evaluation metrics to determine the value of the approaches could capture interactions focused on health information seeking between older adult patrons and library staff, usage of the ICTs such as the Portal at libraries, training modules for library staff on how to use and connect older adults to ICT resources, and changes in health-related information provision skills among library staff (Table 2).

**Table 2:** KT strategies for librarians and libraries

Theme: *Connecting to information via people and venues*	
Subtheme	Suggested KT strategies
Librarians and libraries as knowledge hubs	**Educational outreach and materials**
	• Create and disseminate training modules (or other educational outreach) to familiarize public librarians with ICTs like the Portal, and how to guide and support target users to find and, if necessary, print desired health information • Distribute accessible information pamphlets on various health topics at libraries
	**Interactive small group sessions**
	• Use libraries as a venue for information sessions for patients
	**Champions/opinion leaders**
	• Develop relationships with Librarian Associations (e.g., Canadian Library Association)
	**Conferences**
	• Attend library association meetings or conferences

#### Subtheme: Family and informal caregivers

Family members were common sources of health information and support among the personas: *“He tried to use a computer but without much success, so he relies on his daughter for any advice and questions he has about medicine.*” *(Jack)*. Getting trusted and reliable information to these knowledge users is important. Personas relied on offspring, spouses, and other relatives, as well as friends and caregivers in their communities. Recommended KT strategies include small group sessions facilitated by peers, creating resources for family and other informal caregivers and making them available in primary care offices, and developing public campaigns to increase awareness of these evidence-based sources of information (Table 3).

**Table 3:** KT strategies for family and informal caregivers

Theme: *Connecting to information via people and venues*	
Subtheme	Suggested KT strategies
Family and informal caregivers are common sources of health information	**Educational outreach and materials**
	• Develop and distribute KT resources to be provided by primary care (before the patient or family leave the office) to address informational needs especially during wait times or between appointments
	**Interactive small group sessions**
	• Develop training program(s) for patients and their informal caregivers (family members, friends and neighbours of patients) on how to access and use health information on ICT like the Portal • Support a seminar series or other speaking opportunities for users (older persons, caregivers) to share their experiences with others
	**Media campaign**
	• Purchase ads on websites and social media platforms that patients and their caregivers are likely to visit
	**Champions/opinion leaders**
	• Engage patients and ICT users (older persons, caregivers) in sharing knowledge about the ICT with their peers

#### Subtheme: Community organizations as a source for health information

Personas went to a range of community organizations and locations for information. For example, church was an important community for Mabel: “*…[She] also go[es] to the church and ask[s] fellow parishioners.*” and Alice was *“actively engaged in her church and thinks that the local health department could be more involved in sharing health information with the congregation.”*

Community centres were identified as another common venue for acquiring information. For example, Sally *“…goes to her community recreation centre for booklets and about programs and finds out information … from her friends.”* Similarly, Sandra “*…belong[s] to the [local seniors’ centre] …She’s contacted the…[seniors centre] to get more speakers to talk about many of these medical information [sic]…about learning about medical issues through medical information technology and what resources are available for seniors”.*

Other venues that were mentioned included coffee shops, malls, organizations like the Heart and Stoke Foundation of Canada, and local hospital waiting rooms. KT strategies in the community could target community centres as avenues for passive dissemination of brochures or small group education sessions for community service providers and patrons of the centres (Table 4).

**Table 4:** KT strategies for general public and community organizations as the knowledge users

Theme: *Connecting to information via people and venues*	
Subtheme	Suggested KT strategies
Community organizations are a source for health information	**Educational outreach and materials**
	• Provide or sponsor information pamphlets or sessions for targeted promotion of ICT during senior-related activities • Distribute ICT promotional materials and pamphlets to community recreation centres
	**Media campaign**
	• Broaden dissemination to seniors’ centres, community organizations, other community services • Request to host links to the ICTs on websites of applicable organizations
	**Champions/opinion leaders**
	• Engage with trusted professional and community health information providers (e.g., family health clinics, recreation centres, community health centres, patient health associations) to share consistent information about the ICT within these venues

#### Subtheme: Multiple avenues and formats to seek health information

Persona scenarios illustrated that people use multiple avenues (people, venues, and formats) to seek health information; the process is dynamic and multifaceted. For example, Joe uses multiple information seeking approaches:

*Joe usually uses several ways of getting trusted information: from his family physician…the public library – the librarian helps him access information from the computer…He generally relies on his son, Mark, who is computer literate. Also, he goes to [the] mall to speak with his good friends from [his previous industrial workplace].*

Similarly, another scenario identified multifaceted information seeking approaches which also highlighted the need for consistency in information provided across sources:

*Alice talked about getting information at her doctor’s office or at the library and sometimes from her daughter and sometimes on a website, and the need for that to be similar or else that could be confusing. Or her pharmacist as well… her GP is sort of the next person.*

These illustrate that there are multiple opportunities to support their knowledge seeking needs.

### Theme 2: Health information delivery formats

Users of digital sources want recognizable, trusted sources and preferred resources created in multiple formats. Participants described various print formats in which older adults preferred to access health information, which included magazines, pamphlets, and books:

*June does not look at electronics, but she will look at ads and flyers and pamphlets. So, for example, if Shoppers Drug Mart has a flyer that says this is good for something, she will often think that that is true and purchase that product. (Pete’s wife and caregiver, June).*

Another scenario emphasized the value of health information available in printed formats that are *“just one page, maybe double-sided, and it has a very concise, summarized information and all that she needs on that one page.” (Sally).*

Older adults or the people they trusted also consulted online resources: *“One of her friends told her about the McMaster Portal to check that out. She also had… another friend [who] told her about the Mayo Clinic website.” (Sandra)*, though they identified concerns about trustworthiness. For example, in the case of Pete’s wife and caregiver June:

*…her sister might be able to access electronic information through websites on Google… to be a completely trustable website it should have some kind of government or organization endorsing it…It would also be trustable if it’s the physician or pharmacist who gave June’s sister or June that information or website.*

The various information seeking behaviours and different needs raised by participants suggest that multi-modal KT tools and formats are needed (Table 5). From the scenarios, we see that older adults use the internet to find health information, are interested in recognizing trusted sources, and prefer resources in multiple formats.

**Table 5:** Suggested formats for KT strategies to meet the needs of older adults seeking health information

Theme: *Health information delivery formats*
**Educational outreach and materials**
• Have handouts, poster boards, service demonstrations and sign-ups, or presentations on various relevant health topics at social gathering places such as Bingo halls, churches, community recreation centres, malls, crosswalks, coffee shops • Engage trusted sources (primary care office, Ministry of Health) to provide push notifications to patients regarding healthy aging, common health conditions, the Portal as an example of a trusted source and how to recognize one • Create a shortlist of consumer publications (e.g., magazines, newspapers, community papers, health columns) where adapted versions of Portal-style evidence syntheses could be presented • Send query letters to publishers to explore possibility of publishing Portal-style evidence syntheses as consumer health content • Convert evidence summaries to different formats such as booklets, fact sheets, flyers, newspaper
**Media campaign**
• Targeted ads and information for older adults (geographically or through their affiliation with health condition-based groups on social media platforms) • Increased social media-based awareness and outreach • Navigation to the Portal or other ICT Facebook page should be found in several ways (and should not require searching by the full website name on Facebook or though Google) • Advertisements on billboards or city bus screens • Links to other trusted information sources on the ICT • Work with hosts of other reputable health websites visited or consulted by target users (e.g., Health Canada, Heart and Stroke Foundation, Mayo Clinic) to post cross-endorsements of each other’s content
**Champions/opinion leaders**
• Engage community and health organizations in increasing knowledge or awareness among the public (e.g., at a religious centre, senior centre, community centre, or via a community organization such as the Canadian Association for Retired Persons)

## Discussion

Using findings from a persona-scenario methodology, we have interpreted the narratives of older adult information seeking and searching behaviours and translated them into a set of potential KT strategies to share and promote health information resources to various knowledge users (Table 6). By considering older adults as our knowledge users, deploying strategies informed by the questions of *where* and *from whom* and *in what formats* they are seeking information could increase uptake of health promoting ICTs like the Portal and also other nondigital information resources. A range of approaches have been presented [34], some directly engaging older adults (e.g., small group education sessions) while others indirectly target older adults through the people or places where older adults seek health information. The Portal exemplifies the types of evidence-based health-promoting ICTs that our findings and recommendations are applicable to, but the elements can be generalized to other health information resources aimed at older adults. As suggested by the literature and based on evidence of efficacy, our recommendations promote reliance on multiple KT activities and strategies, which are more effective than single strategies [34, 40].

**Table 6:** Potential outputs of the recommended KT strategies

Knowledge users	Potential strategies and products
Health practitioners and professionals as the knowledge users	Publicity campaign targeted to primary care providers and pharmacy
	Printed materials (pamphlets, brochures, postcards)
	Scripted emails for distribution by providers to patients
Librarians and libraries as the knowledge users	Training modules for library staff on the use of the Portal
	Printed materials (pamphlets, brochures, postcards)
	Small-group education sessions
	Conference booth or presentation
Family and informal caregivers as the knowledge users	Printed materials: pamphlets, brochures, postcards
	Small-group education sessions or training modules for patients or caregivers
	Training program or seminar series co-presented by peers and/or public users
	Advertising campaign through social media
General public/community organizations as the knowledge users	Advertising campaign, print, social media, and other websites
	Printed materials (pamphlets, brochures, postcards)
	Small-group education sessions for service providers
	Information sessions led by trusted individuals for older adults
Delivery formats suitable for older adults	Advertising campaign, print, social media, and other websites
	Printed materials (pamphlets, brochures, postcards)
	Small-group education sessions for service providers

### Knowledge brokers as facilitators for information seeking

Knowledge brokers are intermediaries, frequently between researchers and knowledge users such as policy and decision makers; they effectively make connections between people to facilitate the use of evidence [19, 41–43]. Key elements of knowledge brokering include creating, acquiring, assessing, adapting, applying, and disseminating knowledge, linkage and networking, and enhancing capacity [19, 41]. A strong theme from our findings is of people in various roles essentially acting as knowledge brokers to support the information seeking behaviours of older adults—people well placed to become champions of evidence-based resources. Our findings that older adults rely on their physicians, pharmacists, librarians, friends, and family members are consistent with other studies [13]. Many of the people engaged by older adults could be considered informal knowledge brokers, outside the realm of researchers and health system decision-makers, but nonetheless guiding information seekers and users to appropriate sources of knowledge to support their health and wellbeing. An understanding of the context for KT strategies and the stakeholder relationships involved within and across organizations is essential for successful KT [44]. Providing training and informational resources to knowledge brokers, as suggested by several of the KT strategies, would effectively develop their knowledge brokering competencies [45]. For example, by increasing librarian knowledge and access to training and support, information about the Portal could potentially reach more library visitors. Given that patrons seek out the help of a librarian, librarians could confidently guide clients to more reliable sources of information. While ICTs such as the Portal facilitate the acquisition, assessment, and adaptation of sound research evidence, informal brokers are active within their social groupings and could facilitate dissemination, linkages, and capacity building by exchanging informational support and recommendations among peers within their own networks [46]. They frequently take on the role of surrogate seekers, seeking accurate online health information and communicating it to others to influence healthcare decisions [47].

Receiving information from the identified knowledge brokers enhances the potential use of the resource; older adults prefer health information received from people they trust (physicians, pharmacists, family and friends) than from non-human sources (internet or news) [13]. This was echoed in the information seeking behaviours in the scenarios. A strength of the Portal is the reliability of the information and the trustworthiness of the source [48]. This key message should be highlighted in the KT strategies.

There are inequities in access to health information through the internet across socioeconomic and education levels [5, 6, 8] which were reflected in some of the personas in our study having access to ICTs while others did not. An ongoing survey of health information seeking by older adults in USA (Health Information National Trends Survey [HINTS]) shows that many respondents searched the internet first, though this was more common among younger adults with higher social economic status and those with higher internet skills [8]. The Portal visitors tend to be older, well-educated, retired women in good health [49]. Providing access and educational supports in community settings, such as libraries and community centres, can support engagement among a broader population of older adults and address inequities in access and health and internet literacy.

### KT plan desirability, feasibility, and viability

When developing each of the suggested KT strategies and outputs, the elements of desirability, feasibility, and viability were considered together [50]. The most promising KT strategies where those that met all three elements. In contrast, some suggested strategies, like provision of training for library staff, may be very desirable as they are important knowledge brokers, but the implementation of such a strategy may not be feasible or viable to sustain. These considerations need to be balanced to determine the best approaches. Perhaps education modules targeted to those library staff who run seniors programming may be a more feasible and viable approach. Organizations who wish to share health information resources with older adults will need to consider these three elements within the context of community needs and preferences, organizational scope, and available resources when developing their KT plans.

### KT plan evaluation

Key to any KT plan, the implementation of any of the suggested strategies and outputs should be evaluated [34]. Though many of the strategies are passive forms of dissemination, some actions such as small group education sessions in community settings led by knowledge brokers are likely to produce greater knowledge gains by participants [51]. We have not included detailed evaluation plans here as the suggested strategies should inform context specific KT plans. We hope that the identification of KT strategies allows organizations to build on this exploratory research, by providing theoretical support for development of multi-faceted programs to increase the reach of evidence-based information products to older adults. Evaluation of such programs would follow.

### Strengths and limitations

The study was conceived and designed to generate findings that were not limited to the Portal or even to reliable online health information products; thus, the results are generalizable to other information resources for older adults, to support their health information seeking behaviours as well as searching for and application of the information. Participants were not limited in describing information seeking behaviours in the context of the Portal or online formats, and the personas had a range of technical confidence, ages, and health conditions, which supports transferability to other similar older adult populations. However, we did not achieve maximal variation in sampling a full range of perspectives given that most participants had postsecondary education and access to technology from home; indeed, this was not an objective for this initial project whose primary aim was to generate design specifications. Therefore, due to the limited breadth in perspectives and profiles of participants, transferability to populations not captured here should be done with caution.

The persona-scenario method used in this study has advantages over other more traditional design approaches that rely on interviews or focus groups with pre-defined questions to collect user perspectives as it allows for more participant creativity in defining user interactions, making it a more open approach for identifying user requirements that may not otherwise be considered by a design team [29, 31, 33]. When used in designing the Health TAPESTRY program, the persona-scenario method resulted in 406 design-related products, of which 45 % were novel to the research team [30]. This method therefore has a track record of generating novel design-relevant insights when used with older adults. In our case, it became apparent from the personal scenarios that from who and where personas sought health information was an important theme to capture and could inform KT activities.

Our study included eighteen older adults, which is a reasonable sample size for qualitative design-based research of this nature [52]. We performed a brief member check at the end of scenario sessions through verbal summaries of cross-cutting issues. We did not, however, conduct a member check with the KT strategies proposed in this report, which were derived by the researchers from the themes and content from the persona-scenarios. Although all participants lived in southern Ontario, their personas represented a range of older adults, capturing some rural and urban, male and female, and immigrant perspectives. Though all participants were English-speaking, one scenario reflected the perspective of a child as the caregiver for an older non-English speaker.

## Conclusions

Our findings underline the importance of people, venues, and formats in the actions of older adults seeking trusted health information. These findings highlight the need for enhanced KT strategies to meet their needs across personal and professional networks. KT outputs and activities could include educational outreach/materials, decision support tools, small group sessions, publicity campaigns, champions/opinion leaders, and conferences.

## Supplementary Information

**Additional file 1.**

## Data Availability

The datasets used and/or analysed during the current study are available from the corresponding author on reasonable request.

## Abbreviations

KT: Knowledge translation
ICT: information and communication technologies
