# Supplementary material for: Knowledge translation strategies for sharing evidence-based health information with older adults and their caregivers: findings from a persona-scenario method
Source: BMC Geriatr. 2021 Nov 23;21:665. doi: 10.1186/s12877-021-02588-x (PMC8611832; doi:10.1186/s12877-021-02588-x)
Supplement: Supplementary file 1 — Additional file 1. [file 12877_2021_2588_MOESM1_ESM.pdf]

Appendix: Persona-Scenario Discussion Guide

Study Title: Co-design and production of a user-optimized communication toolbox

for delivering digital health information to older adults: **Phase One**:

Appendix 1: Story Development

This research involves the Health Information Research Unit at McMaster University (HiRU) and the Aging, Community and Health Research Unit (ACHRU).

HiRU creates and shares trusted health information using technology for older adults.

We need your help to improve how we can best provide trusted health information that is relevant to people like you!

- Health information should be relevant to you. It can provide the best ways to look after your health or the health of a loved one.
- You can get health information in many ways, such as: websites, emails, text messages and many other ways.

We want your help to understand how older adults like you prefer to get health information. This will help us design better ways of presenting and delivering it to you. Your help can also increase the usefulness and relevance of health information for older adults in the general public.

Today we want you to think about how you get health information. How could it be best delivered?

Based on your knowledge and experience as an older person or someone caring for an older person, you will create a story. You will work with a partner. First create an imaginary (but believable) lead character. Then start to develop the

story around the character. The story should address a situation in which your character finds and uses health information. The questions below will help you create your story. At the end, you and your partner will share your story with the larger group.

Step 1: Create your character. (15 minutes)

Your lead character will be an older person or their loved one who is looking for health information. He or she may use technology to get information or not. He or she might involve another character to help him or her get the information.

When you create your character give him or her some personality. **Consider these questions as a guide.**

**Character**	
1. name, age, and gender;	
2. level of education;	
3. usual ways of getting trusted health information;	
4. comfort with technology;	
5. situation related to hearing, visual, or other impairments, e.g., disability, or mobility issues;	
6. situation in terms of people involved in his or her care;	
7. living context?	

8. Other: For example: Occupation, hobbies etc.	

Step 2: Create at least one story. (45-60 minutes)

Now create your story. Your character begins to look for trusted health information about staying healthy as he or she ages. Create at least one short story for your lead character who may or may not interact with technology to access this health information.

Consider these questions as a guide to create your story:

Story #1	
1. What problem caused your character to want to look for trusted health information?	
2. How does your character usually get health information? (a computer, a phone, printed material)?	
3. How did your character start to look for trusted health information for this situation?	
4. Thinking of the information resources (including electronic technology) that your character may use**,** consider the following:<br>a) What kinds of non-electronic (e.g., book, flyer, health professional) or<br>electronic resources (e.g., computer, smart phone, tablet) do they use?<br>Where do they access them (e.g., home, library, a relative's house, somewhere else)?<br>b) How did he or she find out about this resource?<br>c) How did he or she start to find health information using this resource to solve the problem?	

d) Did your character get the health information him or herself or was it provided automatically? Describe how this happened.<br>e) Describe the format or the delivery of health information. What does your character see, hear, feel or touch to get health information?<br>f) If your character has any physical challenges, such as vision, hearing, or mobility challenges, how does your character overcome them to access the health information?<br>g) How much health information is there and how is it presented? For example, are there key points, summaries, lists of other resources, or other ways information is presented?<br>h) Does your character need help in using this resource (especially if it's electronic technology) to find the information? If yes, what kind of help does he or she get and from whom?	
5. Is privacy a concern in accessing health information in this situation? How so and how does it affect how you get access to information?	
6. Are there other older persons, caregivers, or other characters in your story? What are they doing when your character is dealing with the problem? Are they also involved? If so, how?	
7. Does your character provide feedback on their experience in accessing this information or the content? How does this happen?	
8. How does the problem get resolved? What happens as a result of interacting with the health information (e.g., what decisions, emotional responses, or other actions happen)?	

Step 3: Report back to larger group a summary of your character and story. (10-15 minutes per small group)

Your discussions and presentations will be audio-taped to ensure that we do not miss anything important from your stories. The audio-tapes will be transcribed and analysed. Your names will not be used.

Step 4: Feedback on the character-story process

We want to thank you for your help today. You have provided us with important ideas about how to best communicate health information to older adults. We will use everyone's ideas to develop a few ways of sharing health information to meet older adults' needs. Once this is done, we may invited you to participate again. We will ask you if you think the way we hope to deliver health information will fit well to address the needs of aging Canadians.

Finally, we would like to take a few minutes to ask you individually for some feedback about your experiences today. If you are willing, we have a short survey for you to complete. You can with answer the survey yourself or we can through it with you verbally. One of the note takers will fill in your answers.

Thank you for your valuable input into the study!
